# Assessing the impact of low-technology emanators alongside long-lasting insecticidal nets to control malaria

**DOI:** 10.1098/rstb.2019.0817

**Published:** 2020-12-28

**Authors:** Joel Hellewell, Ellie Sherrard-Smith, Sheila Ogoma, Thomas S. Churcher

**Affiliations:** 1 MRC Centre for Global Infectious Disease Analysis, Department of Infectious Disease Epidemiology, Imperial College London, Norfolk Place, London W2 1PG, UK; 2 Ifakara Health Institute, Biomedical and Environmental Thematic Group, PO Box 53, Ifakara, Morogoro, United Republic of Tanzania

**Keywords:** emanators, *Anopheles*, *Plasmodium*, long-lasting insecticidal net, outdoor biting, residual transmission

## Abstract

Malaria control in sub-Saharan Africa relies on the widespread use of long-lasting insecticidal nets (LLINs) or the indoor residual spraying of insecticide. Disease transmission may be maintained even when these indoor interventions are universally used as some mosquitoes will bite in the early morning and evening when people are outside. As countries seek to eliminate malaria, they can target outdoor biting using new vector control tools such as spatial repellent emanators, which emit airborne insecticide to form a protective area around the user. Field data are used to incorporate a low-technology emanator into a mathematical model of malaria transmission to predict its public health impact across a range of scenarios. Targeting outdoor biting by repeatedly distributing emanators alongside LLINs increases the chance of elimination, but the additional benefit depends on the level of anthropophagy in the local mosquito population, emanator effectiveness and the pre-intervention proportion of mosquitoes biting outdoors. High proportions of pyrethroid-resistant mosquitoes diminish LLIN impact because of reduced mosquito mortality. When mosquitoes are highly anthropophagic, this reduced mortality leads to more outdoor biting and a reduced additional benefit of emanators, even if emanators are assumed to retain their effectiveness in the presence of pyrethroid resistance. Different target product profiles are examined, which show the extra epidemiological benefits of spatial repellents that induce mosquito mortality.

This article is part of the theme issue ‘Novel control strategies for mosquito-borne diseases’.

## 1. Introduction

Long-lasting insecticidal nets (LLINs) and indoor residual spraying (IRS) with insecticides are effective and widely used methods of controlling malaria [1]. These tools are restricted to targeting mosquitoes inside the home [2]. Mosquitoes that bite people when they are outdoors contribute to residual malaria transmission, which is transmission that remains when there is universal coverage of effective LLINs and IRS [3]. The low force of infection required for ongoing transmission means that even low levels of outdoor biting may be sufficient to prevent current indoor-based interventions from interrupting transmission in many places [4,5]. New methods of controlling outdoor biting mosquitoes are urgently needed to support the drive for malaria control and elimination.

Emanators are spatial repellents that passively release low concentrations of airborne insecticides to reduce human and mosquito contact. Their primary aim is to create a vector-free space, though they may also disrupt the feeding process by interfering with host-seeking and blood-feeding behaviour and could reduce long-term mosquito survival [6]. There are a variety of home-made and commercial emanators under development which differ in complexity and in their imagined use-case [7,8]. Here, we focus specifically on low-technology, passive emanators (hereafter referred to simply as ‘emanators’) which can be assembled with relative ease by soaking strips of hessian cloth in the volatile pyrethroid transfluthrin before attaching them to a metal frame [9]. This prototype has been shown to be effective even against pyrethroid-resistant mosquitoes and can then be placed outside homes [9] or in bars [10], reducing the number of mosquito bites on people who are in close proximity.

Emanators have been evaluated in a field trial in Tanzania by measuring the number of mosquitoes attempting to feed on volunteers sitting up to 40 m away from fully operational, or control, devices [9]. For users who sat immediately next to a device, emanators averted between 71 and 91% of bites from *Anopheles arabiensis* mosquitoes. Additionally, there was decreasing but still significant protection for volunteers who sat up to 5 m away. There was no observable increase in the number of mosquitoes attempting to feed on volunteers who sat 80 m away from the emanator, suggesting that bites prevented by the emanator were not immediately redirected to non-users who sat nearby. Emanators have been evaluated indoors, outdoors and in semi-field tunnels [11], though their full entomological impact and the extent to which they induce mosquito mortality in a particular environment remains unclear [12–14].

Realization of the epidemiological significance of residual transmission and the threat posed by mosquitoes developing resistance to the insecticide used in LLINs [15] means that there is increased investment in the development of outdoor interventions. It remains unclear how effective this new class of vector control tool is when used at scale, how they may interact with existing control interventions and what entomological characteristics should be prioritized in the development process. Here, we use a mathematical model of malaria [5,16,17] to predict how emanators, in combination with LLINs, could reduce malaria transmission. Entomological data from the passive transfluthrin emanator used in the Tanzanian field trial and other target product profiles are used to investigate how the effectiveness of mass emanator deployment might vary with mosquito bionomics and LLINs with different levels of pyrethroid resistance.

## 2. Methods

### (a) Modelling framework

**Figure 1.**
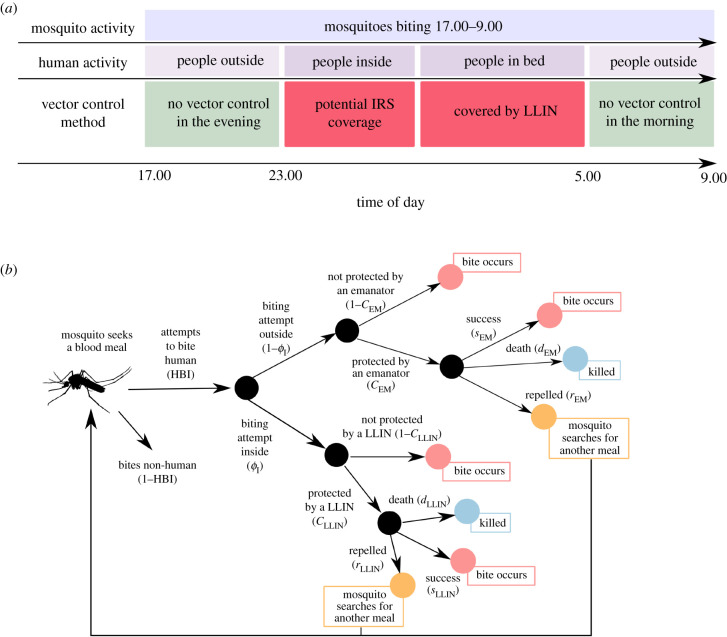
(*a*) Graphical representation of the how LLIN use leaves periods in the morning and evening when mosquitoes are still biting. (*b*) Flowchart of how outdoor biting and emanator use are incorporated into the malaria transmission model structure. Mosquitoes bite indoors or outdoors with a probability depending on the overall proportion of biting attempts that take place indoors (*ϕ*_I_). The outcome of the feeding attempt depends on the proportion of the population protected by a bednet (*C*_LLIN_) or an emanator (*C*_EM_). If there is no intervention, then the mosquito will go on to feed on the host. If there is an intervention present then the outcomes depend on what the intervention is. For emanators, the mosquito can be repelled (exit the area without blood-feeding, *r*_EM_), be killed (*d*_EM_) or successfully bite (*s*_EM_). For LLINs, the mosquito can be repelled (*r*_LLIN_), be killed (*d*_LLIN_) or successfully bite (*s*_LLIN_). Mosquitoes that are repelled go back to the start of the flowchart and can bite outdoors or indoors again with the same probabilities.

Emanators are assumed to be placed outside the home where people congregate in the morning and evening (figure 1*a*). The model is parameterized using mosquito-landing data collected on volunteers sitting outside [9]. Entomological impact is likely to be different inside structures where insecticide vapour concentrations may be greater. For simplicity, it is assumed that emanators remain outside at night and do not directly influence the protection provided to people when they are under bednets. Emanator population coverage, denoted *C*_EM_, is, therefore, the proportion of the population who use an emanator outside their home. The entomological impact of emanators on the likelihood that a blood-feeding mosquito will die, successfully feed, or be repelled and attempt to re-feed, in the presence of emanators and LLINs is outlined in figure 1*b* (following the original conceptual structure of Le Menach *et al.* [18]).

The efficacy of emanators will depend on the proportion of attempted bites that happen outdoors. Following previous notation, let *ϕ_I_* be the proportion of mosquito feeding attempts taken when people are inside the home in the absence of any intervention [5] (for simplicity, herein this is called ‘indoor biting’, which has been referred to as *π*_i_ [4,19] in previous spatial repellent modelling work). This is calculated from hourly estimates of mosquito biting behaviour throughout the night and combined with human data recording the time people go indoors [20]. The degree of outdoor biting in the absence of interventions is, therefore, 1 − *ϕ_I_*. Estimates of outdoor biting are taken from a recent systematic review that measured human behaviour and mosquito biting rates indoors or outdoors using human landing catches for the main malaria vectors in sub-Saharan Africa [21]. Different mosquito species vary in their propensity to bite humans compared with other mammals, typically cattle. The extent of this zoophagy is estimated using blood-meal analysis of wild-caught mosquitoes prior to the introduction of control interventions and is summarized by the human blood index (HBI). If a mosquito attempts to bite a person outdoors using an emanator, then the mosquito has a probability of successfully feeding (*s*_EM_), being repelled away from the user (*r*_EM_) or being killed (*d*_EM_). This is initially calculated from the Ogoma *et al*. study [9], making the assumption that the emanator does not directly kill the mosquito (or impede future refeeding), though the impact of induced-mortality is investigated later.

The model assumes that the same population of mosquitoes bites people indoors and outdoors, and that a repelled mosquito will go on to search for another meal with the same chances of success as before (i.e. *C*_EM_, *C*_LLIN_ and *ϕ_I_* are independent of feeding time). This means that, each time, a repelled mosquito will attempt to feed outdoors with a probability of 1 − *ϕ_I_*, so the overall chance of it biting outside over the feeding cycle will increase if the mosquito is repelled by an indoor intervention (figure 1*b*). In addition to possibly preventing infectious bites on emanator users, being repelled also increases the average time between mosquito feeds (increasing their chance of dying from other non-emanator causes) and reduces the mosquito biting rate on humans. If mosquitoes bite inside then they interact with LLINs as previously specified [5], where they have a chance of successfully feeding, dying or being repelled. Previous modelling work has suggested low mosquito HBI can increase emanator impact as prevented bites are more likely to be on animals rather than another human [22,23]. Two levels of mosquito anthropophagy are, therefore, investigated: an *Anopheles gambiae* sensu stricto-like mosquito with high HBI (HBI = 92%) and an *A. arabiensis*-like mosquito with low HBI (HBI = 16%).

### (b) Emanator efficacy

Evidence suggests emanators show a clear pattern of decreasing efficacy the further the user is away from the device [9], since the concentration of insecticide in the air is likely to be highest nearest to the source. People are unlikely to remain in close proximity to their emanator at all times, so their protection will depend upon both: (i) how effective the emanator is at different distances and (ii) how much time people spend at different distances from an emanator.

To estimate (i) emanator efficacy, we use data from Ogoma *et al.* [9]. We fit the following exponential decay function *E*(*x*) to the proportion of bites prevented by an emanator at a given distance:

2.1 $$\begin{array}{c} E(x)=A{\text{e}}^{-Yx}, \end{array}$$

where *x* is the distance from the emanator in metres, *Y* is the rate at which emanator efficacy decays with distance and *A* is the proportion of bites prevented at 0 m. The function was fitted to data from Ogoma *et al.* [9] using least-squares regression and assessed for goodness of fit using the *R*^2^ linear regression measure.

Estimating (ii), the time spent in close proximity to a device, is more challenging. Despite searching, we are not aware of any datasets that measure the distance that people are from their houses in the evenings (or other places that emanators might be used) in Tanzania. How much time people spend near their homes is likely to vary by income, the hours of work available nearby, age and weather—therefore, datasets may be localized in space and time. In the absence of these data, it seems reasonable to also approximate the time spent at each distance from an emanator at the population level by an exponential decay function. Let *m* be the median distance a user is from an emanator when outdoors (i.e. the probabilities that a person is further from or closer to their emanator than *m* metres are both equal, 50%). This could either be a single emanator or the minimum distance to an emanator when multiple devices are deployed (though we assume the user only receives the benefit of the closest device). It is assumed that the device behaves identically for each person in the case that multiple people use the same device. The distribution of time spent near an emanator (when people are outside, assumed to be the same across the population), denoted *D*(*x*), is defined as

2.2 $$\begin{array}{c} D(x)=\lambda {\text{e}}^{-\lambda x}, \end{array}$$

where *λ* is chosen to give a desired median value of *m*:

2.3 $$\begin{array}{c} \lambda =\frac{m}{\ln(2)}\;. \end{array}$$

It is assumed that people's proximity to emanators when they are outdoors is independent of the time of day (i.e. people are not more likely to be sitting close to an emanator nearer to midnight when mosquito abundance may be greater). If this is the case, then the two distributions, *E*(*x*) and *D*(*x*), are then multiplied and summed up over all distances to give the proportion of bites prevented by an emanator used in the model, *r*_EM_:

2.4 $$\begin{array}{c} r_{\mathrm{EM}}=\int_{0}^{\infty }D(x)E(x)\;. \end{array}$$

### (c) Implementation scenarios

**Figure 2.**
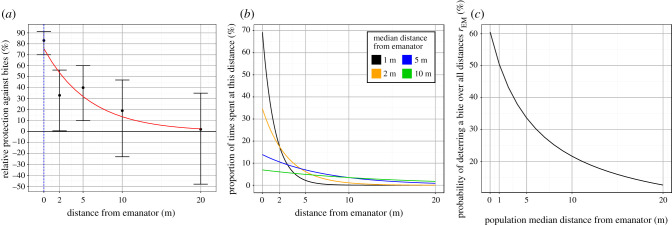
(*a*) The emanator efficacy at reducing biting on a person at different distances as observed in the Tanzanian study. Black points correspond to the percentage reduction in human landing catches on volunteers (vertical black lines indicate corresponding 95% confidence interval estimates) while the red line shows the fitted continuous exponential function *E*(*x*). (*b*) Four theoretical examples of the (exponential) distribution that represent the population median distance from their emanator, be it 1 m (black line), 2 m (yellow) 5 m (blue) or 10 m (green). (*c*) Overall estimates of emanator efficacy taking into consideration the time taken close to a device. Black line shows the proportion of bites averted by the emanator over all distances (*y*-axis) for a given median population distance from an emanator (*x*-axis) and the emanator efficacy outlined in (*a*).

The impact of emanators was explored by predicting the public health impact of the combined distribution of emanators and LLINs compared with distributing LLINs alone. This reflects the current use-case scenario since emanators are not intended to replace LLINs but to help achieve elimination in settings where there is good LLIN coverage. The regularity at which emanator efficacy declines over time is unclear, so for simplicity we assume that devices are replaced/re-dipped sufficiently regularly that the efficacy remains constant. We simulated scenarios where emanators and LLINs or LLINs alone were given to 80% of the population (with LLINs distributed at years 0, 3 and 6). Malaria elimination was defined as a reduction in all-age slide prevalence by microscopy below 1% for 50 consecutive days. We also assume that people spent the majority of their time outside near their emanator by choosing a median population distance of 1 m from the emanator (figure 2*b*). Outdoor biting is estimated from the proportion of the population outdoors over the course of the day (electronic supplementary material, figure S1a) and the proportion of bites taken outside at this time (estimated from human landing catches [4]; electronic supplementary material, figure S1b). To investigate a range of scenarios, it is assumed that outdoor biting will vary from 0 to 50% which is within the observed range of 0–87% (with a median of 13%) estimated by reanalysing results of a recent meta-analysis [21] (electronic supplementary material, figure S1c). Each scenario was repeated with mosquitoes parameterized for the high and low HBI.

To explore how pyrethroid resistance changes the interaction between emanators and LLINs, the level of pyrethroid resistance in the mosquito population was varied (as defined by the percentage of mosquitoes that would survive a discriminating dose bioassay test). The level of pyrethroid resistance influences LLIN efficacy by reducing the mortality effect and duration of protection [24]. Emanator efficacy is assumed to be independent of the level of bioassay survival since it is unclear how pyrethroid resistance changes the efficacy of the device (i.e. mosquitoes can survive prolonged LLIN contact but are repelled away from emanator users). In the Kilombero Valley in southern Tanzania, where the emanator study used to parameterize the model took place, 58 and 66% of local mosquito populations survived discriminating dose bioassays for permethrin and deltamethrin, respectively, in June 2015 [25]. Despite this reasonably high level of resistance, there was still significant bite prevention recorded for emanator use when the emanator study took place during two phases in 2012–2013 and 2014–2015. A deterministic version of a widely used compartmental model describing the transmission dynamics of malaria in humans and mosquitoes is used as parameterized previously [5,24]. All source code and a full list of parameters are provided in the electronic supplementary material.

## 3. Results

### (a) Emanator efficacy

The emanator prototype characterized from the field data is predicted to avert 76% of bites on people immediately beneath a device (at a zero distance), dropping to approximately 50% of bites averted by 2.5 m (figure 2*a*). Observed data broadly follow the chosen exponential distribution. The efficacy of an emanator varies depending on how close people are to a device. Figure 2*b* shows four different theoretical human behaviour patterns as regards the distance spent away from an emanator with a median of 1–20 m. For example, if people spend their time outdoors a median of 1 m away from an emanator, the model assumes that 80% of their time is within 2 m. In this scenario, the emanators would be predicted to avert approximately 60% of all attempted bites (figure 2*c*). Since emanators are most effective at close range, the proportion of bites prevented across all distances decreases as people spend more of their time further away from the emanator. Emanators would still avert just over 20% of outdoor bites if the population median distance from their emanators was 10 m.

**Figure 3.**
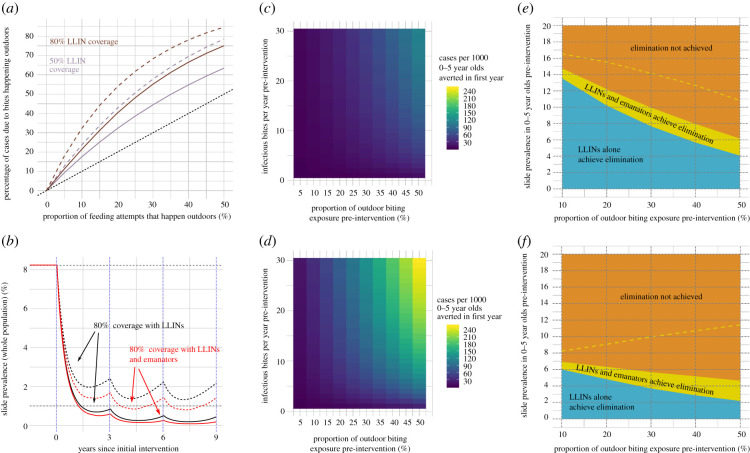
(*a*) The relationship between the proportion of outdoor biting (in the absence of any intervention) and the proportion of cases across the whole population due to bites happening outdoors (post-intervention) for two different LLIN coverages, 80% (brown) and 50% (purple). The effect is shown for mosquitoes with a high HBI (solid lines) and a low HBI (dashed lines). Dotted black line shows where equivalence between the two proportions would be. This value is estimated by comparing the numbers of clinical cases in two scenarios, one with observed level of coverage and the other where all outdoor biting is prevented. (*b*) An example of the intervention scenario where LLINs or LLINs plus emanators are distributed to 80% of the population every 3 years at years 0, 3 and 6. Again, solid lines represent mosquitoes with high HBI (92% of bitting attempts are taken on humans) and dashed lines with a low HBI (16%). It is assumed that 20% of biting happens outdoors prior to any intervention and there is no pyrethroid resistance. (*c*) The number of additional cases averted per 1000 0–5 year olds per year when emanators are distributed alongside LLINs for varying pre-intervention entomological inoculation rates (*y*-axis) and proportions of outdoor biting (*x*-axis). Mosquitoes are assumed to have a high HBI and full susceptibility to pyrethroids on LLINs. (*d*) As for (*c*), except for mosquitoes with a low HBI. (*e*) The maximum pre-intervention slide prevalence in 0–5 year olds at which LLINs alone (blue) or LLINs and emanators (yellow) can achieve elimination within 9 years (*y*-axis) and how this changes with the proportion of outdoor exposure in the absence of any intervention (*x*-axis). The dashed yellow line shows how an ‘optimal’ emanator that prevents all outdoor biting performs in addition to LLINs. Mosquitoes are assumed to have a high HBI and full susceptibility to pyrethroids on LLINs (*f*) Repeat of (*e*) but with mosquitoes with a low HBI.

As LLIN coverage increases, outdoor biting becomes more epidemiologically important as a larger proportion of cases of malaria will result from bites taken outside (figure 3*a*). For mosquitoes with a high HBI (92%), 10% outdoor biting (prior to introduction of LLINs) and 80% of people using an effective LLIN, the model predicts just over 20% of clinical cases occur from bites received outside (figure 3*a*). This rises to just over 30% for mosquitoes with a low HBI. This higher increase for mosquitoes with a low HBI (16%) is because the absolute amount of transmission declines (as mosquitoes failing to feed on humans go on to bite non-humans) so the proportion of all exposure that occurs outdoors increases as mosquitoes become less anthropophagic. These dynamics are driven by the interaction between the pre-intervention outdoor biting exposure and the HBI influencing the level of outdoor biting exposure in the presence of interventions (this is explored in detail including graphical illustrations in electronic supplementary material, figures S2 and S3).

### (b) Epidemiological impact

LLINs in an area with susceptible mosquitoes are predicted to be highly effective at reducing malaria prevalence (figure 3*b*). Providing people using bednets with an emanator further reduces disease prevalence, but the added benefit is relatively low in comparison with the impact of nets. Nevertheless, distributing this type of emanator on top of LLINs is still predicted to prevent between 0.44 and 122 cases per 1000 0–5 year olds in the first year of distribution against mosquitoes with high HBI. More cases are prevented in areas with high outdoor biting and greater transmission intensity (figure 3*c*). Emanators also prevented more cases when deployed against mosquitoes with a low HBI, preventing between 1.45 and 255 cases per 1000 0–5 year olds (figure 3*d*).

To illustrate how different factors will impact emanator efficacy, we examine how different LLIN and emanator use is likely to eliminate the disease. For areas with 10% pre-intervention outdoor biting exposure, including emanators alongside LLINs increases the maximum pre-intervention prevalence (in 0–5 year olds) at which elimination could be achieved from 13.5 to 14.8%. The likelihood of elimination decreases with more outdoor biting as fewer mosquitoes are killed by the bednet (figure 3*e*). Interestingly, in this scenario, the added benefit of emanators remains roughly constant as increasing outdoor biting causes a greater loss in the effectiveness of LLINs than any corresponding increase in the effectiveness of emanators (which is explored in more depth in electronic supplementary material, figure S3). This is because the emanator simulated here is assumed to only partially averts bites and importantly does not induce mortality like LLINs. Similarly, the likelihood of disease elimination decreases when mosquitoes have a lower HBI (for a given malaria prevalence, figure 3*f*) as zoophagy reduces the likelihood a mosquito comes into contact with a bednet and dies.

Comparing the real emanator used in a realistic scenario where people spend a percentage of their time close to the device with a hypothetical ‘optimal emanator’ where the devices prevent all outdoor biting shows the maximum effect that a strongly repellent emanator can achieve (dashed yellow line, figure 3*e*,*f*). When 50% of pre-intervention biting exposure takes place outdoors, LLINs alone achieved elimination when the pre-intervention prevalence was below 4.1%, adding optimal emanators increased this to 10.8%. Using the optimal emanator against a less anthropophagic mosquito population (16% of bites are taken on humans rather than 92%) reveals different mechanisms driving impact as elimination is more likely with increased outdoor biting (figure 3*f*). This is because optimal emanators prevent all outdoor bites, so more and more mosquitoes are feeding on alternative hosts as outdoor biting increases, lowering disease transmission.

**Figure 4.**
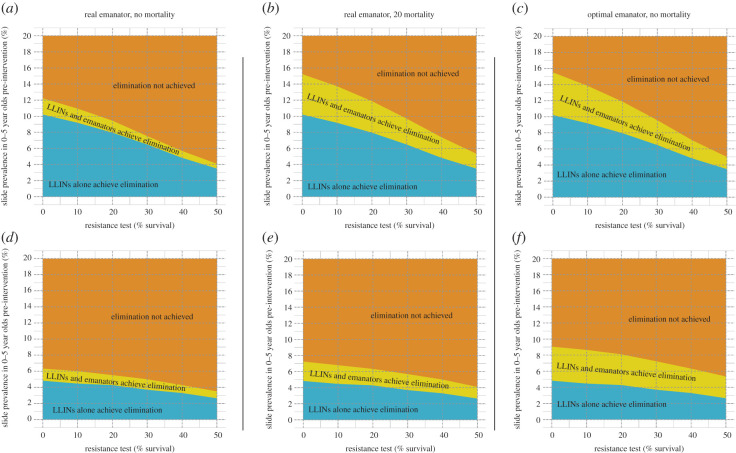
(*a*) How the maximum pre-intervention prevalence in 0–5 year olds at which elimination is achievable (*y*-axis) decreases with an increasing pyrethroid resistance (defined as the proportion of mosquitoes that survive a discriminating dose bioassay, *x*-axis). The mosquito has a proportion of outdoor biting (pre-intervention, fixed at 20%) and a high HBI (HBI = 92%). (*b*) A scenario where emanators now have a 20% mortality effect that is retained regardless of the level of bioassay survival. (*c*) A scenario involving the optimal emanator that repels all outdoor biting mosquitoes but does not cause any mortality. (*d*–*f*) Repeats of (*a*–*c*), but with a mosquito that has a much lower HBI (16%). It is assumed that 20% of biting happens outdoors prior to any intervention in all of the panels in this figure.

In many countries in sub-Saharan Africa, resistance to pyrethroids on LLINs is becoming prevalent in mosquito populations [26]. Currently, any emanators that would be distributed are likely to be used alongside pyrethroid-only LLINs, which are less effective at killing mosquitoes than they have been in the past. While it remains unclear to what extent resistance confers protection against the actions of an emanator, the transmission model can be used to examine how the additional benefit of emanator distribution changes as LLINs become less potent. Strikingly, when used against mosquitoes with a high HBI, the additional benefit of emanator distribution also declines as pyrethroid resistance increases (figure 4*a*). This further suggests that the mechanism through which emanators cause most of their impact (when used alongside working LLINs) is through repelling outdoor biting mosquitoes (that have a high HBI) away from people in the evening, making them more likely to bite indoors where they are, in the absence of resistant mosquitoes, more likely to be killed by a LLIN. As LLINs become less able to kill mosquitoes, this effect diminishes and so the additional benefit of emanators is smaller. For mosquitoes with a weaker preference for biting humans, the pre-intervention prevalence at which elimination can be achieved is lower across the board (figure 4*d–f*). However, the additional effect of emanators does not diminish as clearly along with the level of bioassay survival. This reflects the fact that mosquitoes are less likely to bite another human once repelled, now repellence is more comparable to mortality since a mosquito is unlikely to attempt to bite a human again.

It is unclear whether volatile pyrethroids released by emanators outdoors induced mosquito mortality. Nevertheless, here we investigate the impact of mortality to determine whether it should be part of a target product profile. For the high HBI mosquitoes, if 20% of mosquitoes coming into contact with emanators are killed (regardless of the level of knockdown resistance), then this would drastically increase the effectiveness of emanators and LLINs used together (figure 4*b*). Alternatively, if the repelling effect of an emanator is increased to 100% (as in the optimal emanator explored above), this would cause roughly the same public health impact as the 20% mortality effect (figure 4*c*). For low HBI mosquitoes, the optimal emanator (figure 4*f*) is more effective than an emanator that induces mortality (figure 4*e*), which again shows how repelling mosquitoes that are unlikely to try and bite a human again becomes more akin to having killed them in terms of epidemiological outcomes.

## 4. Discussion

The public health benefit of adding emanators or spatial repellents to communities protected by insecticide-treated bednets depends heavily on the bionomics of the local mosquito population. The mathematical modelling exercise indicated that emanators provided an additional benefit over LLIN use alone in all explored scenarios. Generally, this benefit was largest in areas that where the mosquito population had a low HBI, high outdoor biting and were susceptible to the insecticides used on LLINs, suggesting that spatial repellents could be targeted to locations with specific entomological characteristics. The work also reiterates that emanators that induce mosquito mortality should be prioritized.

Simulations indicate that for mosquitoes with a high HBI, the biggest epidemiological impact of emanators is to push bites away from people outdoors in the evening and onto another vector control method that kills mosquitoes. This means that, even if emanators continue to repel pyrethroid-resistant mosquitoes, their effectiveness will diminish along with the mortality effect of LLINs. In these scenarios, emanators are unlikely to counteract the loss of LLIN effectiveness through the rise of pyrethroid-resistant mosquitoes [24] though they could be effectively paired with non-pyrethroid IRS or LLINs with alternative insecticides, which would still induce mortality in mosquitoes repelled away from emanators. This mode of action is less important for mosquitoes with a low HBI as people will be protected by the emanator deflecting mosquitoes onto non-human hosts, and any mosquitoes resting indoors, or deflected to rest indoors, can be killed by the IRS. Models indicate that up to twice as many cases of malaria might be averted by adding emanators to LLINs in areas with low HBI, in part because bednets are less effective in these situations and there is currently very ineffective control. Repelling zoophagic mosquitoes can often be as effective as killing them, since they will be unlikely to bite humans twice over the course of their lifespan. This result is in line with previous modelling work that has shown that the advantage of a mortality effect over a repellent effect reduces as HBI decreases [22]. Understanding where emanators and spatial repellents will have the greatest impact will depend on the interaction between the entomological impact of the product and the setting (degree of outdoor biting, HBI, effectiveness of existing control interventions). For example, an emanator that purely repels mosquitoes could provide the greatest impact in areas with high outdoor biting (figure 3*c*,*d*), though a product that induces mortality may be more effective in areas with low outdoor biting (figure 4*b*,*e*) where they have a higher chance of directly killing the mosquito.

The effectiveness of emanators, as parameterized in this transmission model using data from the low-cost emanator from Ogoma *et al*. [9], certainly warrants their inclusion in a package of vector control tools aiming to reduce outdoor residual transmission. The scale of their public health impact is predicted to be relatively modest relative to that of effective LLINs, so ensuring high population coverage of LLINs should remain a priority. Nevertheless, the number of cases averted by emanators is still substantial and the lack of current, viable alternatives for targeting outdoor transmission together with the low cost of emanators means that they are likely to be highly cost-effective relative to other anti-malarial interventions being trialled [27].

While these modelling results are encouraging, there remain aspects of emanators (and other spatial repellents) that require a better understanding to inform predictions of impact. Malaria mosquito models differ in their structure and parameterization [28–30], reflecting the uncertainty in human–mosquito interactions and with consequences for public health predictions. For example, the plasticity in when and where mosquitoes bite would alter the impact of emanators, since a mosquito that prefers outdoor biting (and is not merely reacting to vector control coverage) would keep trying to bite outside and would not be pushed onto LLINs. In addition to mosquito behaviour, human behaviour will modify how effective emanator distributions are: in terms of both compliance (staying close enough to the emanator for it to be effective) and exposure (being outdoors in the evening and morning). Data currently exist that delineate between people being in bed and indoors, but they do not record when people spend time away from the house completely. Since current emanators are only effective within several metres, to investigate impact further, more data would be needed on how close people stay to them and how this varies with their age and other factors such as occupation. In addition to this, emanator effectiveness will likely be altered by local climatic conditions such as wind speed and direction, ambient air pressure and humidity. These factors would vary geographically as well as temporally over the course of an evening.

Further clarification of the entomological effects of emanators is also required. Firstly, there is the challenge of detecting the modes of action of emanators when used outdoors. Spatial repellents used in experimental hut trials show a range of modes of action, including mortality, confusion, feeding inhibition and reduced egg laying [31,32]. However, the magnitude and severity of the modes of action of airborne pyrethroids are likely to be dependent on insecticide concentration, so it cannot be assumed that the effects indoors will translate to outdoor use. Ideally, models should be parameterized using information on the concentration and duration of insecticide exposure experienced by free-flying mosquitoes over their feeding cycle in different environments where emanators might be used. This can then be combined with data on how this period of exposure influences mosquito mortality, blood-feeding and other sublethal factors. As we have shown, if outdoor concentrations of airborne pyrethroids could cause mortality then this would greatly increase emanator effectiveness. If this does happen then this analysis likely underestimates emanator impact. Inducing relatively modest mortality in the low-cost emanator had a similar epidemiological impact to preventing all outdoor bites, suggesting killing ability should be prioritized in any emanator or spatial repellents target product profiles. Secondly, it is also important to consider how these modes of action might change, given pyrethroid resistance and whether mosquitoes are suffering sublethal morbidity after exposure [33,34]. Finally, it is also important to consider how mosquito mortality caused by emanators may contribute to selection pressure for pyrethroid-resistant mosquitoes. If emanator-caused mortality does select for increased knockdown resistance, then perhaps higher repellent properties are preferable.

Combinations of push–pull interventions have been highly effective when using repellents and mosquito traps [35,36]. This work highlights the need for care when deciding how interventions that repel mosquitoes should be combined with LLINs, which are likely to remain the mainstay of malaria control; repelling mosquitoes away from effective LLINs is likely to reduce overall mosquito mortality, impeding control. Epidemiological trials of mosquito repellents (which include topical repellents and insecticide-treated clothing) used in combinations with LLINs show mixed results [37]. Variation in the patterns of use of repellents could help to account for the lack of observable additional effect in some trials. Focusing research on the interactions of novel and existing tools will be important to improve predictions of impact.

## Supplementary Material

Supplementary figures

## Data accessibility

Data on the effectiveness of emanators at different differences (figure 2*a*) can be accessed from [9]. Data on mosquito feeding behaviour and human behaviour leading to outdoor biting exposure (electronic supplementary material, figure S1a,b) can be accessed from [21].
