# Supplementary material for: Assessing the impact of low-technology emanators alongside long-lasting insecticidal nets to control malaria
Source: Philos Trans R Soc Lond B Biol Sci. 2020 Dec 28;376(1818):20190817. doi: 10.1098/rstb.2019.0817 (PMC7776935; doi:10.1098/rstb.2019.0817)
Supplement: Supplementary figures [file rstb20190817supp1.docx]

**Supporting Material - Assessing the Impact of Low-technology Emanators Alongside Long-lasting Insecticidal Nets to Control Malaria**

**Authors:** Joel Hellewell^1^, Ellie Sherrard-Smith^1^, Sheila Ogoma^2^, Thomas S. Churcher^1^

^1^ MRC Centre for Global Infectious Disease Analysis, Department of Infectious Disease Epidemiology, Imperial College London, Norfolk Place, London, W2 1PG, UK.

^2^ Ifakara Health Insititute, Biomedical and Environmental Thematic Group, PO Box 53, Ifakara, Morogoro, United Republic of Tanzania

1. **Calculating estimates of the proportion of outdoor exposure pre-intervention**


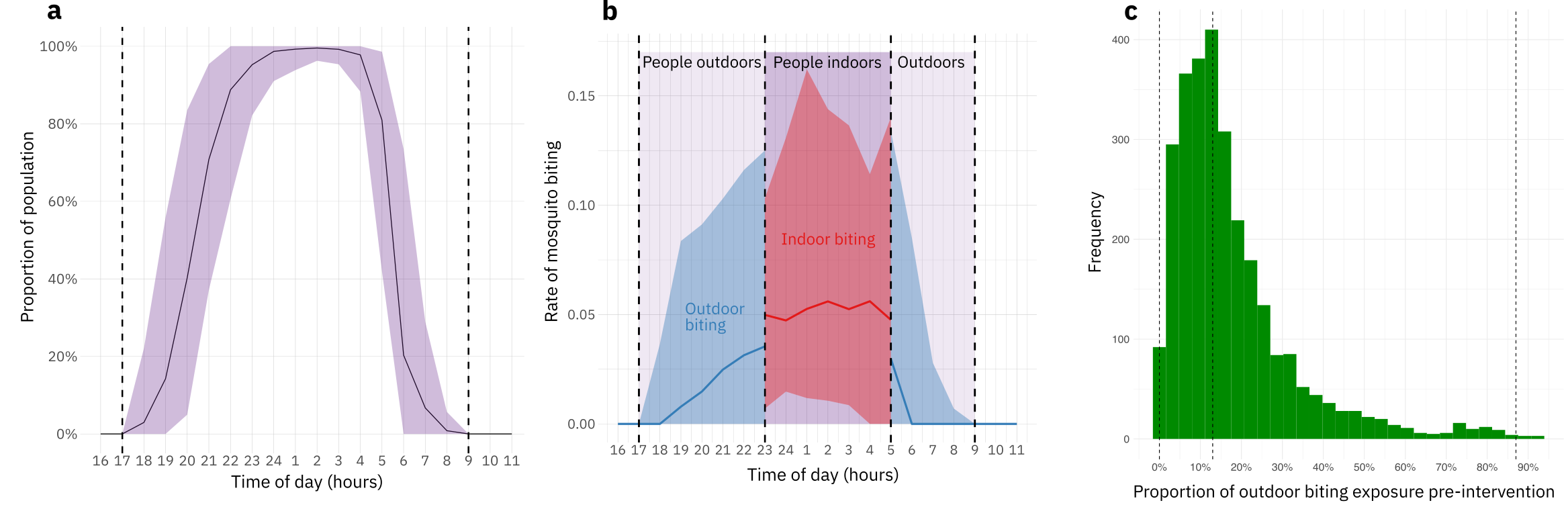


**Figure S1:** a) The median proportion (black line) and 95% intervals (purple) of the population that are indoors from all studies is shown for each hour of the day as estimated by a systematic meta-analysis (Sherrard-Smith et al 2019). b) Median and 95% intervals for the observed outdoor and indoor biting rates from all studies. c) The range of estimates for the proportion of mosquitoes which bite outside prior to the introduction of control interventions (refered to in main text as outdoor biting, 1 - ϕ_I_) that are generated from bootstrapping random combinations of the range of different observed human (a) and mosquito (b) behaviours. See Sherrard-Smith et al 2019 for full details data and calculations used

1. **How mosquito anthropophagy interacts with the proportion of clinical cases due to outdoor biting exposure**


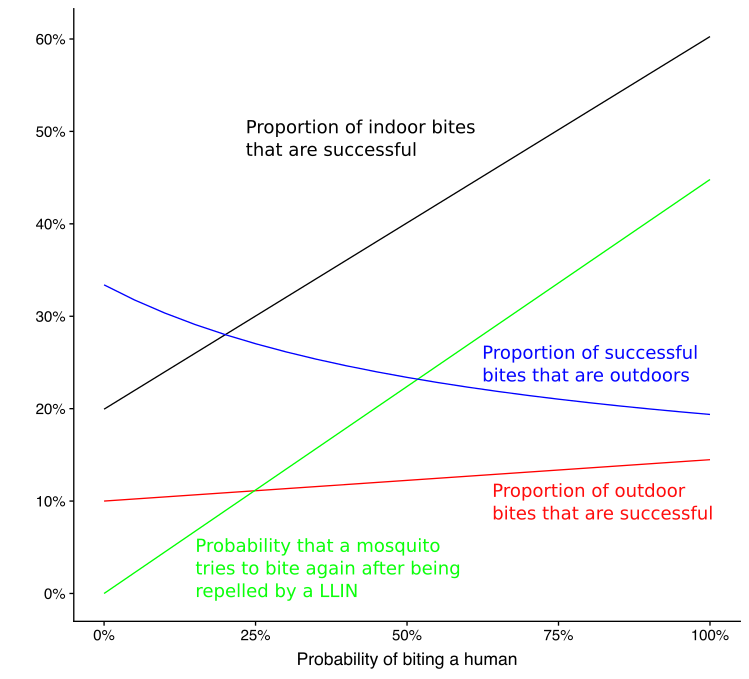


**Figure S2:** How the human biting index (HBI) changes the proportion of clinical cases due to outdoor biting exposure in a population where 80% of the population use bednets and 90% of pre-intervention biting exposure occurs indoors. Firstly, as the probability of biting a human decreases, the probability that a mosquito tries to bite again decreases. This is because repelled mosquitoes become more likely to go and feed on an animal instead since their preference for feeding on humans has become less strong. This has different impacts on indoor and outdoor biting.

The proportion of bites that are successful, given that they are indoors, decreases as HBI decreases. The mosquitoes that were trying to feed repeatedly were very likely to try and bite again indoors, now that they are not trying to feed again the net just has to repel or kill the mosquito once, which it has quite a good chance of doing.

The proportion of bites that are successful, given that they are outdoors, also decreases as HBI decreases, but not by anywhere near as much as for indoor biting. This is because few of the mosquitoes that were trying to feed again when HBI is high were trying to bite again outside. Now that they are no longer trying to feed again, there is only a small impact on outdoor biting success (note there are no emanators in this scenario).

The blue line shows the proportion of bites that are outdoors, given that they are successful. A higher proportion of successful bites happen outdoors when HBI is high because of the relative changes in the success of biting indoors or outdoors. This explains how, in Figure 3a, a higher proportion of clinical cases are the result of outdoor biting when the human biting index is lower.

1. **How pre-intervention outdoor biting exposure effects emanator impact**


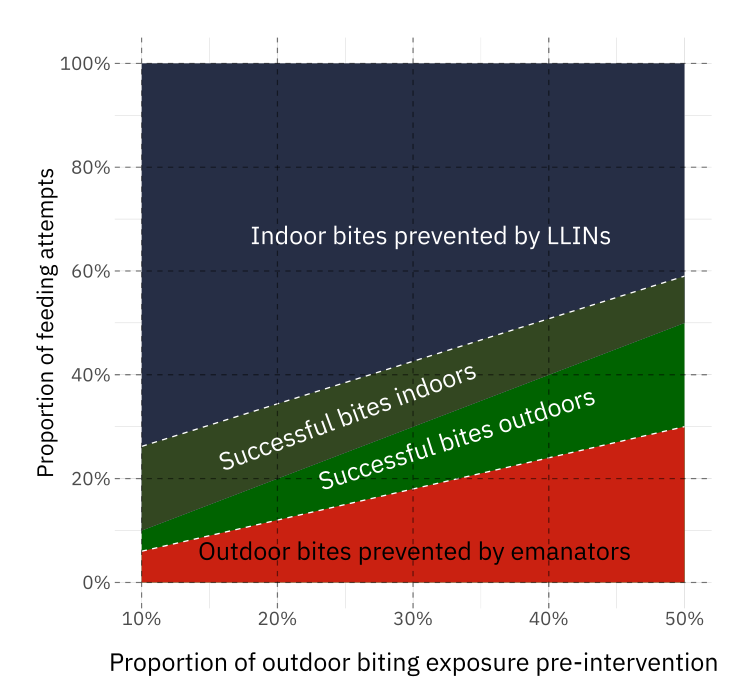


**Figure S3:** Figure shows how the post-intervention location that attempted bites happen and the likelihood of their success changes as more biting occurs outdoors pre-intervention. The proportion of bites that are successful falls in the green areas between the two white dashed lines. As more pre-intervention exposure occurs outdoors there are more outdoor bites prevented by emanators (red area). However, there are also more successful bites outdoors (light green area), as well as fewer bites prevented indoors (dark blue area) and successful bites indoors (dark green area). The proportion of all bites that are successful (the two green areas between the white dashed lines) is greater when there is more pre-intervention outdoor biting. This can be understood in terms of the relative effectiveness of emanators and bednets. When more biting attempts happen outdoors before intervention, the lower effectiveness of the emanator compared to nets means that there are more successful bites overall, even though emanators also prevent a higher proportion of overall bites.

1. **Code for the analysis**

The code to reproduce the figures in this analysis is provided in this R package: <https://github.com/jhellewell14/emanator.model>

It requires this additional package to be installed:

<https://github.com/jhellewell14/ICDMM>

1. **List of model parameters**

A list of model parameters is provided in:

Griffin, J. T. et al. Reducing Plasmodium falciparum malaria transmission in Africa: A model-based evaluation of intervention strategies. PLoS Med. 7, e1000324 (2010).
